# Supplementary material for: Motor context dominates output from purkinje cell functional regions during reflexive visuomotor behaviours
Source: eLife. 2019 Jan 25;8:e42138. doi: 10.7554/eLife.42138 (PMC6374073; doi:10.7554/eLife.42138)
Supplement: Transparent reporting form [file elife-42138-transrepform.docx]

***eLife’s* transparent reporting form**

We encourage authors to provide detailed information *within their submission* to facilitate the interpretation and replication of experiments. Authors can upload supporting documentation to indicate the use of appropriate reporting guidelines for health-related research (see [EQUATOR Network](http://www.equator-network.org/%20)), life science research (see the [BioSharing Information Resource](https://biosharing.org/)), or the [ARRIVE guidelines](http://www.plosbiology.org/article/info:doi/10.1371/journal.pbio.1000412) for reporting work involving animal research. Where applicable, authors should refer to any relevant reporting standards documents in this form.

If you have any questions, please consult our Journal Policies and/or contact us: [editorial@elifesciences.org](mailto:editorial@elifesciences.org).

**Sample-size estimation**

- You should state whether an appropriate sample size was computed when the study was being designed
- You should state the statistical method of sample size computation and any required assumptions
- If no explicit power analysis was used, you should describe how you decided what sample (replicate) size (number) to use

Please outline where this information can be found within the submission (e.g., sections or figure legends), or explain why this information doesn’t apply to your submission:

The total number of Purkinje cells is ~500 in the larval zebrafish, and we record from a) all Purkinje cells in 6 fish with calcium imaging and b) 74 additional Purkinje cells with high-resolution electrophysiology across the cerebellum in order to capture the organization of this population. The number of samples analyzed were based on the number of good preparations produced by our experimental protocols and were not pre-determined by power analysis. We nevertheless tried to use our previous knowledge of noise in physiological signals and variability between individuals to estimate a reasonable sample size prior to experimentation. With this in mind, we required that at least 5 trials (repetitions) were obtained from electrophysiological recordings. The total sample size for each experiments for which data were successfully obtained, given as cells or animals as appropriate, is given in the text and figure legends throughout.

**Replicates**

- You should report how often each experiment was performed
- You should include a definition of biological versus technical replication
- The data obtained should be provided and sufficient information should be provided to indicate the number of independent biological and/or technical replicates
- If you encountered any outliers, you should describe how these were handled
- Criteria for exclusion/inclusion of data should be clearly stated
- High-throughput sequence data should be uploaded before submission, with a private link for reviewers provided (these are available from both GEO and ArrayExpress)

Please outline where this information can be found within the submission (e.g., sections or figure legends), or explain why this information doesn’t apply to your submission:

All experiments were performed on individuals at one time point (no technical replicates were possible for this in vivo work) and samples sizes are indicated throughout the manuscript. Biological replicates were obtained from experiments with individual animals sampled from multiple clutches (e.g. non-siblings) on different days. Number of biological replicates are as follows: Calcium imaging in Purkinje cell (PC) populations, N=6 (Fig 1); calcium imaging in granule cell (GC) populations, N=7 (Fig 6); simultaneous single-cell electrophysiology and calcium imaging, N=5 (Fig S2); electrophysiology in single PCs, N=61 paralyzed (Figs 3-5, 7, S5, S7) and N=13 semi-paralyzed (Fig S7); electrophysiology in single GCs, N=17 (Fig 6); PC morphology, N=50 (Fig 4, S6). No outliers were removed, all data was analyzed. Incomplete data sets (calcium imaging with only partial brain volumes, or electrophysiology recordings with less than 5 trial repetitions) were not included in the analysis. Our data contains no high-throughput sequence data.

**Statistical reporting**

- Statistical analysis methods should be described and justified
- Raw data should be presented in figures whenever informative to do so (typically when N per group is less than 10)
- For each experiment, you should identify the statistical tests used, exact values of N, definitions of center, methods of multiple test correction, and dispersion and precision measures (e.g., mean, median, SD, SEM, confidence intervals; and, for the major substantive results, a measure of effect size (e.g., Pearson's r, Cohen's d)
- Report exact p-values wherever possible alongside the summary statistics and 95% confidence intervals. These should be reported for all key questions and not only when the p-value is less than 0.05.

Please outline where this information can be found within the submission (e.g., sections or figure legends), or explain why this information doesn’t apply to your submission:

Statistical tests are described in the text and figure legends and explained in the methods (see "Quantification and Statistical Analysis"). P-values are reported

throughout the text and figure legends. N is always reported (including, where applicable, N cells from N animals). Raw data is presented where useful (e.g. Fig 3a). All data showing means with error bars or shaded error represent the mean plus/minus standard error of the mean, as reported in figure legends.

(For large datasets, or papers with a very large number of statistical tests, you may upload a single table file with tests, Ns, etc., with reference to sections in the manuscript.)

**Group allocation**

- Indicate how samples were allocated into experimental groups (in the case of clinical studies, please specify allocation to treatment method); if randomization was used, please also state if restricted randomization was applied
- Indicate if masking was used during group allocation, data collection and/or data analysis

Please outline where this information can be found within the submission (e.g., sections or figure legends), or explain why this information doesn’t apply to your submission:

There were no experimental treatments/groups in this study.

**Additional data files (“source data”)**

- We encourage you to upload relevant additional data files, such as numerical data that are represented as a graph in a figure, or as a summary table
- Where provided, these should be in the most useful format, and they can be uploaded as “Source data” files linked to a main figure or table
- Include model definition files including the full list of parameters used
- Include code used for data analysis (e.g., R, MatLab)
- Avoid stating that data files are “available upon request”

Please indicate the figures or tables for which source data files have been provided:

Supplemental videos are provided to show raw calcium imaging data from the Purkinje cell population during stimulus presentation.

Example electrophysiological datasets are available at https://zenodo.org/record/1494071. An example imaging dataset is available at https://zenodo.org/record/1638807.
